# Environmental Fate of Soil Applied Neonicotinoid Insecticides in an Irrigated Potato Agroecosystem

**DOI:** 10.1371/journal.pone.0097081

**Published:** 2014-05-13

**Authors:** Anders S. Huseth, Russell L. Groves

**Affiliations:** 1 Department of Entomology, Cornell University, New York State Agricultural Experiment Station, Geneva, New York, United States of America; 2 Department of Entomology, University of Wisconsin-Madison, Madison, Wisconsin, United States of America; Texas Tech University, United States of America

## Abstract

Since 1995, neonicotinoid insecticides have been a critical component of arthropod management in potato, *Solanum tuberosum* L. Recent detections of neonicotinoids in groundwater have generated questions about the sources of these contaminants and the relative contribution from commodities in U.S. agriculture. Delivery of neonicotinoids to crops typically occurs as a seed or in-furrow treatment to manage early season insect herbivores. Applied in this way, these insecticides become systemically mobile in the plant and provide control of key pest species. An outcome of this project links these soil insecticide application strategies in crop plants with neonicotinoid contamination of water leaching from the application zone. In 2011 and 2012, our objectives were to document the temporal patterns of neonicotinoid leachate below the planting furrow following common insecticide delivery methods in potato. Leaching loss of thiamethoxam from potato was measured using pan lysimeters from three at-plant treatments and one foliar application treatment. Insecticide concentration in leachate was assessed for six consecutive months using liquid chromatography-tandem mass spectrometry. Findings from this study suggest leaching of neonicotinoids from potato may be greater following crop harvest in comparison to other times during the growing season. Furthermore, this study documented recycling of neonicotinoid insecticides from contaminated groundwater back onto the crop via high capacity irrigation wells. These results document interactions between cultivated potato, different neonicotinoid delivery methods, and the potential for subsurface water contamination via leaching.

## Introduction

The neonicotinoid group of insecticides is among the most broadly adopted, conventional management tools for insect pests of annual and perennial cropping systems [1]. Benefits of the neonicotinoid group of compounds include flexibility of application, diversity of active ingredients, and broad spectrum activity [2]. Moreover, growers have readily adopted neonicotinoids for two specific reasons: first, these compounds are fully systemic in plants after soil application and second, several new generic formulations have recently become available which have incentivized their continued use in many crops [1]–[3]. Since 2001, the United States Environmental Protection Agency (EPA) has classified several neonicotinoids as either conventional, reduced-risk pesticides, or as organophosphate alternatives [4],[5]. EPA certification often requires replacement of older, broad-spectrum pesticides with newer, more specific products for management of key economic pests. Critical attributes of replacement insecticides include documented reductions in human and environmental risk when compared to older, broad-spectrum pesticides [5]. Despite acceptance of neonicotinoid insecticides as reduced-risk by growers and regulatory agencies, nearly two decades of widespread, repetitive use has resulted in several insecticide resistance issues, impacts on native and domestic pollinators, and unanticipated environmental impacts [6]–[9].

The environmental fate of several neonicotinoid active ingredients have been previously assessed. Previous studies focused on degradation and movement processes in soil, leachate, and runoff [10]–[15]. The leaching potential of the neonicotinoids into groundwater, as well as persistence in the plant canopy, is related to properties of the chemicals and delivery method of the compound to the crop (Fig. S1)[12],[15],[16]. Soil application (e.g., seed treatment or in-furrow) has been adopted as the principal form of insecticide delivery in potato production as it provides the longest interval of pest control, while also reducing non-target impacts, and limits exposure to workers when compared to foliar application methods. Since 1995, soil-applied neonicotinoids (i.e., clothianidin, imidacloprid, thiamethoxam) have been the most common pest management strategy used to control infestation of Colorado potato beetle, *Leptinotarsa decemlineata* Say; potato leafhopper, *Empoasca fabae* Harris; green peach aphid, *Myzus persicae* Sulzer; and potato aphid, *Macrosiphium euphorbiae* Thomas. The now widespread and extensive use of these systemic neonicotinoid insecticides, coupled with the recent detection of thiamethoxam in groundwater [17],[18], supports the hypothesis that potato pest management may contribute a portion of the documented neonicotinoid contaminants reported in Wisconsin, USA. Furthermore, we hypothesized that neonicotinoid insecticides applied to potato are most vulnerable to leaching in the spring season when the root system of the plant has yet to fully exploit all of the active ingredient applied directly in the seed furrow. Large rain events at this time could drive insecticide leaching from potato and subsequent groundwater contamination at large scales. In this study, we examined how neonicotinoid concentrations in leachate were altered in response to different insecticide delivery methods using potatoes grown under commercial production practices. We also report the patterns of historic neonicotinoid insecticide detections in groundwater using water quality surveys collected by the Wisconsin Department of Agriculture, Trade and Consumer Protection-Environmental Quality Section (WI DATCP-EQ). Second, using potato as a model system, we analyzed leachate captured below different seed treatments, soil-applications, and foliar delivery treatments for thiamethoxam using liquid chromatography-tandem mass spectrometry (LC/MS/MS) over two consecutive field seasons. In this experiment, thiamethoxam was chosen as one representative insecticide in a broader group of water-soluble neonicotinoids. Moreover, this active ingredient represented the majority of positive neonicotinoid detections in groundwater monitoring surveys conducted by the WI DATCP-EQ [17], [18]. Third, using identical quantitative methods, we measured thiamethoxam concentration in irrigation water collected from operating, high-capacity irrigation wells at two time points in each sampling year. And finally, we characterize irrigation use and production trends of crops that may contribute to neonicotinoid detection in groundwater. Results of this study increase our understanding about the influence of insecticide delivery method on the neonicotinoid insecticides leaching from potato into the surrounding environment.

## Materials and Methods

### Ethics Statement

No specific permits were required for the field study described here. Access to field sites was granted by the private landholder to conduct leaching experiments. No specific permissions were needed to present publically available records provided by Wisconsin Department of Agriculture, Trade and Consumer Protection or Wisconsin Department of Natural Resources. Field studies did not involve any endangered or protected species.

### Groundwater Contamination

Permanent groundwater monitoring wells, maintained by the WI DATCP-EQ, were used to measure neonicotinoid contamination of subsurface water resources as one component of an ongoing study documenting agrochemical (e.g., insecticides, herbicides, nutrients) impact on groundwater quality. Beginning in 2006, analytical water quality assessments for neonicotinoid contamination were conducted by the Wisconsin Department of Agriculture Trade and Consumer Protection-Bureau of Laboratory Services. Concentrations of acetamiprid, clothianidin, dinotefuran, imidacloprid, and thiamethoxam were monitored in 20–30 different monitoring well locations from 2006–2012. Presented are positive detections of those insecticides in different monitoring wells from 2006–2012 [17],[18]. Data provided by WI DATCP-EQ characterize the temporal and spatial profile of thiamethoxam and other neonicotinoid detections that occurred between 2008–2012. These data are presented in summary as a foundation for following objectives (Table 1).

**Table 1:** Positive (means±SD) neonicotinoid detections in groundwater from 2008–2012, State of Wisconsin Department of Agriculture Trade and Consumer Protection.

							Insecticide concentration (µg/L)^d^		
Year	County	Area potato (ha)^a^	Row crops (ha)^b^	Percent potato^c^	Well ID	*N* positive samples	clothianidin	imidacloprid	thiamethoxam
2008	Adams	2,617	21,385	10.9	6	2	-	-	4.34 (4.97)
	Grant	0	47,827	0.0	10	1	-	-	1.25
	Iowa	18	25,795	0.1	11,12,13	9	-	-	1.50 (0.67)
	Richland	29	9,582	0.3	16	1	-	-	0.69
	Sauk	30	31,931	0.1	17	2	-	-	2.41 (1.32)
	Waushara	2,630	29,447	8.2	20	2	-	-	0.67 (0.05)
2009	Adams	3,989	24,894	13.8	6	2	-	-	5.31 (5.12)
	Dane	22	101,527	0.0	9	1	-	-	1.61
	Iowa	343	33,375	1.0	11,12	3	-	-	1.31 (0.68)
	Richland	87	14,402	0.6	16	1	-	-	1.26
	Sauk	328	40,571	0.8	17	2	-	-	3.00 (0.94)
2010	Adams	4,188	24,871	14.4	6	4	3.43	-	2.97 (2.04)
	Brown	1	39,322	0.0	7	1	-	-	0.52
	Dane	34	110,979	0.0	8,9	4	0.54 (0.24)	0.54	1.08
	Grant	49	74,566	0.1	10	1	0.73	-	-
	Iowa	356	38,840	0.9	11,12,13	7	-	-	1.25 (1.02)
	Sauk	188	45,309	0.4	17	5	0.41	-	1.81 (0.88)
	Waushara	4,184	33,576	11.1	19,20	2	-	2.77 (0.81)	-
2011	Adams	4,066	27,693	12.8	2,5,6	9	0.63 (0.36)	0.33	0.63 (0.26)
	Brown	7	38,309	0.0	7	1	-	-	0.21
	Dane	33	107,214	0.0	8	2	0.62 (0.19)	-	-
	Grant	13	75,436	0.0	10	1	0.30	-	-
	Iowa	47	40,138	0.1	12	4	-	0.34 (0.09)	0.88 (0.23)
	Portage	7,364	45,324	14.0	15	1	-	-	0.32
	Sauk	213	46,686	0.5	17,18	5	0.54 (0.10)	-	1.92 (0.43)
	Waushara	4,536	36,676	11.0	19,20,21,23	23	0.25 (0.03)	0.78 (0.69)	1.40 (0.56)
2012	Adams	4,263	27,037	13.6	1,3,4,6	6	0.52 (0.30)	0.51 (0.26)	0.27
	Dane	11	115,501	0.0	8	1	0.67	-	-
	Grant	4	72,920	0.0	10	1	0.26	-	-
	Iowa	369	40,764	0.9	12	2	0.24	0.28	0.44
	Juneau	907	28,542	3.1	14	2	0.42 (0.18)	-	0.20
	Portage	7,622	46,337	14.1	15	2	-	0.47	0.47
	Waushara	5,904	38,999	13.1	21,22,23	13	-	0.68 (0.88)	1.51 (0.72)
			summary	N = 23		67	25	30	68
						Average	0.62 (0.63)	0.79 (0.83)	1.59 (1.51)
						Range	0.21–3.34	0.26–3.34	0.20–8.93

a Acreage estimates generated from USDA National Agricultural Statistics Service – Cropland Data Layer, 2008–2012 [26].

b Row crops class is the sum of the following crop areas (ha): maize, soy, small grains, wheat, peas, sweet corn, and miscellaneous vegetables and fruits.

c Percent potato calculated as the potato area grown annually divided by total arable row crop acreage (other row crops + potato).

d Positive neonicotinoid detections extracted from long-term, groundwater wells maintained by the WI-DATCP-EQ Program.

### Experimental Site and Design

In 2011 and 2012, leaching experiments were conducted 6 km west of Coloma, Wisconsin. Experiments were planted in two different fields approximately 0.5 km apart on 20 May 2011 and 11 May 2012. The soil at both sites consisted of Richford loamy sand (sandy, mixed, mesic, Typic Udipsamments) [19]. Soil composition was 7% clay, 82% sand, and 11% silt. Organic matter was 0.53 percent by weight. Study sites soils had a high infiltration rate (Hydrological Soil Group A), a high saturated hydraulic conductivity (K_sat_) at 28 micrometers per second, and an available water capacity rating of 0.1 cm per cm [19]. No restrictive layer that would impede water movement through the soil has been documented [19]. Study site soil was formed in the bed of glacial Lake Wisconsin from parent material of glacial till overlain by glacial outwash [20]. Upper soil horizons (A and B) are sand with minimal structure. Subsurface soil (C horizon) had no structure. Irrigation pivots in sample fields withdrew water at a depth of 37 m and the water table depth (static water level) was approximately 6 m for both sites [21].

A randomized complete block design with four insecticide delivery treatments and an untreated control was established using the potato cultivar, ‘Russet Burbank’. Plots were 0.067 ha in size and planted at a rate of one seed piece per 0.3 m with 0.76 m spacing between rows. Each year, experiments were nested within a different ∼32 ha commercial potato field, and maintained under commercial management practices by the producer (e.g., nutrient application timing, chemical usage, tillage practices, etc.), with the exception of insecticide inputs. The decision to locate these experiments in commercial fields was, in part, based upon access to a center pivot irrigation system to best duplicate water inputs used to produce commercial potato in Wisconsin. All other inputs and production strategies (e.g. tillage, fumigation, fertility, and disease management) were conducted by the producer with equipment and products in a manner consistent with the best management practices for potato production in Wisconsin. Prior to planting in each season, a tension plate lysimeter (25.4×25.4×25.4 cm) was buried at a depth of 75 cm below the soil surface. Lysimeters were constructed of stainless steel with a porous stainless steel plate affixed to the top to allow water to flow into the collection basin over each sampling interval. Experimental blocks were connected with 9.5 mm copper tubing to a primary manifold and equipped with a vacuum gauge. A predefined, fixed suction was maintained under regulated vacuum at 107±17 kPa (15.5±2.5 lb per in^2^) with a twin diaphragm vacuum pump (model UN035.3 TTP, KnF, Trenton, NJ) connected to a 76 L portable air tank. Each treatment block was equipped with a data-logging rain gauge (Spectrum Technologies, Inc. model # 3554WD1) recording daily water inputs at a five minute interval. Data was offloaded with Specware 9 Basic software (Spectrum Technologies, Inc., Plainfield, IL, USA) and aggregated into daily irrigation or rain event totals using the *aggregate* and *dcast* function in R (package: reshape2, [22]). Irrigation event records were obtained from the grower to identify days and estimated inputs of water application throughout the growing season.

### Insecticides and Application

Thiamethoxam treatments (Platinum 75SG, 75% thiamethoxam per formulated unit, Syngenta, Greensboro, NC) were selected to represent a common, soil-applied insecticide in potato. A second formulation of thiamethoxam was selected to represent a common pre-plant insecticide seed treatment in potato (Cruiser 5FS, 47.6% thiamethoxam per formulated unit, Syngenta, Greensboro, NC). Each insecticide formulation is used to manage early season infestations of Colorado potato beetle, potato leafhopper, and colonizing aphid in Wisconsin potato crops. Commercially formulated insecticides were applied at maximum labeled rates for in-furrow (140 g thiamethoxam ha^−1^) and seed treatment (112 g thiamethoxam ha^−1^ at planting density of 1,793 kg seed ha^−1^) for potato [23]. A calibrated CO_2_ pressurized, backpack sprayer with a single nozzle boom was used to deliver an application volume of 94 liters per hectare at 207 kPa through a single, extended range, flat-fan nozzle (TeeJet XR80015VS, Spraying Systems, Wheaton, IL) for in-furrow applications. Spray applications were directed onto seed pieces in the furrow at a speed of one meter per second and furrows were immediately closed following application. Seed treatments were applied using a calibrated CO_2_ pressurized backpack sprayer with a single nozzle boom delivering an application volume of 102.2 L per hectare at 207 kPa through a single, extended range, flat-fan nozzle (TeeJet XR80015VS, Spraying Systems, Wheaton, IL) was used for delivery of thiamethoxam in water (130 mL) directly to suberized, cut seed pieces (23 kg) 24 hours prior to planting. Seed treatments were allowed to dry in the absence of light at 20°C during that pre-plant period. A novel soil application method, impregnated copolymer granules, was included as another treatment in an attempt to stabilize applied insecticide in the soil. Polyacrylamide horticultural copolymer granules (JCD-024SM, JRM Chemical, Cleveland, OH) were impregnated at an application rate of 16 kg per hectare. The polyacrylamide treatment was included as a novel delivery method to stabilize insecticide in the rooting zone and possibly reduce leaching in the early season. Thiamethoxam (0.834 g, Platinum 75SG) was initially diluted in 250 mL of deionized water and 100 µL of blue food coloring was incorporated into solution to ensure uniform mixing (brilliant blue FCF). Insecticide solutions were mixed with 75 g polyacrylamide then stirred until the liquid was absorbed and a uniform color was observed. Impregnated granules were vacuum dried in the absence of light for 24 hours at 20°C. Treated granules were divided into even quantities per row and evenly distributed into the four treatment rows for each polyacrylamide plot. A single untreated flanking row was planted between plots. All soil-applied insecticides were applied on 20 May 2011 and 11 May 2012 at the time of planting.

Two foliar applications of thiamethoxam (Actara 25WG, 25% thiamethoxam per formulated unit, Syngenta, Greensboro, NC) sprayed on the same plot were included as a fourth delivery treatment. Two successive neonicotinoid applications are recommended for foliar control of pests in potato [23]. Foliar thiamethoxam was applied using a calibrated CO_2_ pressurized backpack sprayer delivering an application volume of 187.1 liters per hectare at 207 kPa through four, extended range flat-fan nozzles (TeeJet XR80015VS, Spraying Systems, Wheaton, IL) spaced at 45.2 cm. The first foliar application was followed approximately seven days later with a second equivalent rate of thiamethoxam to total the season-long maximum labeled rate (105 g thiamethoxam ha^−1^) [23] and were timed to coincide with the appearance of 1^st^ and 2^nd^ instar larvae of native populations of *L. decemlineata*. Foliar applications of thiamethoxam were applied on 28 June and 5 July in 2011 and 15 and 22 June in 2012. Although total amounts of active ingredient differ by formulation, these rates are identical to registered label recommendations [23] and reflect the maximum amount of active ingredient used on an average hectare of cultivated potato. Specific chemical properties of formulated thiamethoxam that affect solubility and leaching potential in soil can be found in Gupta et al. [15] and the references therein (Fig. S1).

### Chemical Extraction and Quantification

Lysimeter leachate was sampled twice monthly beginning on June 1 of each year and concluding in October of 2011 and November of 2012. Total leachate volume was recorded for each plot. A 500 mL subsample was taken from each plot into a 0.5 L glass vessel and immediately placed on ice and refrigerated at 4–6°C in the laboratory prior to analysis. Samples were homogenized into a 400 mL monthly (i.e., two samples per month) sample as percent volume per volume dependent on total catch measured in the field. Neonicotinoid residues from monthly water samples were extracted using automated solid phase extraction (AutoTrace SPE workstation, Zymark, Hopkinton, MA) with LiChrolut EN SPE columns (Merk KGaA, Darmstadt, Germany). If visual inspection of sample found excessive sediment contamination, samples were filtered through a 0.45 µm filter prior to extraction. Columns were conditioned prior to extraction with 3 mL of methanol (MeOH) and 3 mL of water. 210 mL of sample were loaded onto columns and rinsed with 10 mL of water then dried under flowing nitrogen for 15 minutes (N-evap, Organomation, Berlin, MA). Samples were eluted using a 50% ethyl acetate (EtOAc) and 50% methanol solution to collect a 2 mL sample fraction. Sample extract fractions were analyzed using a Waters 2690 HPLC/Micromass Quattro LC/MS/MS (Waters Corporation, Milford, MA). All thiamethoxam residues were identified, quantified, and confirmed using LC/MS/MS by the Wisconsin Department of Agriculture Trade and Consumer Protection-Bureau of Laboratory Services. The method detection limit (MDL) of the extraction procedure was 0.2 µg L^−1^.Specific conditions for all quantitative procedures follow WI-DATCP Standard Operating Procedure #1009 developed from Seccia et al. [24] and references therein.

### Irrigation Use and Crop Area

To determine the extent of irrigated agriculture present within the watershed, we utilized current high capacity well pumping data and irrigated agriculture estimates derived from digital imagery. Publically available operator reporting data for high capacity agricultural pivots were obtained from the Wisconsin Department of Natural Resources Bureau of Drinking Water and Groundwater. Records included location information and pumping volume for the year 2012. High capacity wells service several irrigated fields and often these fields are further divided into individual crop management units each with unique irrigation requirements. We digitized the area watered by all identifiable center pivot, linear move, and traveling gun irrigation systems using digital aerial photography to measure the total number of management units present within the greater Central Wisconsin Water Management Unit watershed [25] (ArcGIS version 10.1, Redlands, CA). Fields were subdivided into management units using the consistent divisions in crop types with a sequence of National Agricultural Statistics Service Cropland Data Layer (NASS-CDL) [26] thematic data and aerial photography images [25] from 2010–2012.

To determine agronomic trends in the Central Sands vegetable production region of Wisconsin, we used a combination of publically available land use data and current neonicotinoid registration information. A geospatial watershed management boundary layer delineated by the Wisconsin Department of Natural Resources [27] was used to generally define the spatial extent where agriculture could be contributing to the detection of neonicotinoid insecticides in subsurface water. The Central Wisconsin Water Management Unit extent was used to estimate annual crop composition using the NASS-CDL [26] from 2006–2012 using ArcGIS. From these data, we selected major crops that frequently receive either seed or in-furrow soil-applied neonicotinoid insecticide treatments. Application rates were identical for several similar crops (e.g. soybean and green bean), and so, we chose to aggregate crops based on insecticide rate and crop type into three primary groups: maize, beans, and potato [23],[28]–[30]. These crop groups comprise the majority of production area in the Central Wisconsin Water Management Unit extent. To our knowledge, limited information exists documenting the proportion of different soil-applied neonicotinoid active ingredients that are used on a per crop basis in the Central Wisconsin Water Management Unit. Based on this level of uncertainty, we chose not to extend tabulated crop areas to a direct calculation or estimate of neonicotinoid active ingredients applied.

### Data Analysis

To determine the impact of different insecticide delivery treatments on thiamethoxam leachate detected over time, we reported the mean concentration over a period of several months. All lysimeter analyses included samples where neonicotinoid insecticides were not detected (i.e., zero detections). All data manipulation and statistical analyses of leachate concentrations were performed in R, version 2.15.2 [31] using the base distribution package. Functions used in the analysis are available in the base package of R unless otherwise noted. Observed concentration for time points in each year were subjected to a repeated-measures analysis of variance (ANOVA) using a linear mixed-effects model to determine significant delivery (i.e. treatment), date, and delivery×date effects (*P*<0.05). Because the agronomic conditions differed between years and given that our comparison of interest was at the insecticide delivery treatment level, insecticide concentrations were analyzed separately for each year. Mixed-effects models (i.e., repeated-measures analysis of variance) were fit using the *lme* function (package nlme, [32]). Empirical autocorrelation plots from unstructured correlation model residuals were examined using the *ACF* function (package nlme, [32]). Correlation among within-group error terms were structured and examined in three ways: first, unstructured correlation, second, with compound symmetry using the function *corCompSymm* and third, with autoregressive order one covariance using the function *corAR1* (package nlme, [32])[33]. Since models were not nested, fits of unstructured, compound symmetry, and autoregression order one covariance were compared using Akaike’s information criterion statistic with the function *anova* (test = “F”). Data were transformed with natural logarithms before analysis to satisfy assumptions of normality, however untransformed means are graphically presented. In 2012, a single lysimeter in the polyacrylamide treatment of the leachate study malfunctioned and these observations were dropped from subsequent analyses leading to an unbalanced replicate number for that treatment (N = 3) in 2012. Water input data collected from tipping bucket samplers were averaged across block by day and aggregated as cumulative water inputs using the *cumsum* function. All summary statistics and model estimates were extracted using *aggregate*, *summary*, and *anova* functions.

## Results and Discussion

### Groundwater Detections

**Figure 1:**
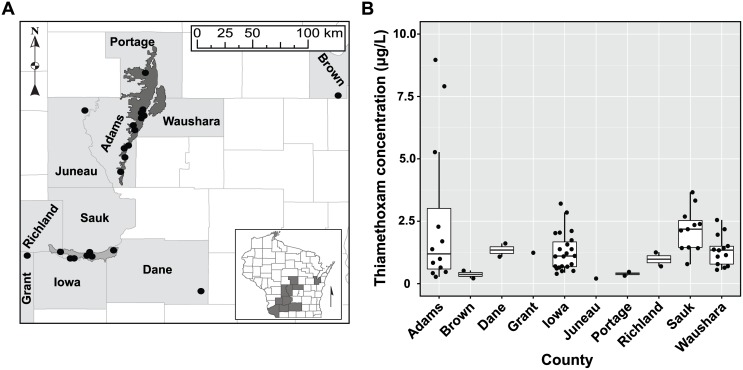
Positive thiamethoxam residue detections in groundwater 2008–2012. Points in the map (A) correspond to positive detection locations. Dark grey shaded region indicates the Central Sands potato production region. Light grey delimits the Lower Wisconsin River potato production region. Positive detections were obtained from established agrochemical monitoring wells collected by the Wisconsin Department of Agriculture, Trade and Consumer Protection (DATCP)-Environmental Quality division in collaboration with the Wisconsin DATCP Bureau of Laboratory Services. Boxplots (B) indicate average concentration detected from 2008–2012. Points show individual measured concentrations.

Neonicotinoid insecticides were detected at 23 different well monitoring well locations by WI-DATCP-EQ surveys between the years 2008 and 2012 (Table 1). These annual surveys, administered by WI-DATCP-EQ, occur at sensitive geologic or hydrogeologic locations that are at high risk of non-point source agrochemical leaching. Specifically, two agriculturally intensive production regions of the state, the Central Sands and Lower Wisconsin River valley, are classified as high-risk areas for groundwater contamination and are frequently monitored for the presence of common agrochemicals (Fig. 1A). These regions have well-drained, sandy soils and easily accessible groundwater for irrigation that has driven agricultural intensification focused on vegetable production. Commercial potato is a key component in the agricultural production sequence, but is also rotated with many other specialty crops such as: carrots, onions, peas, pepper, processing cucumber, sweet corn, and snap beans. Unfortunately, the unique soil and water characteristics supporting a profitable specialty crop production system are also particularly vulnerable to groundwater contamination with water-soluble agricultural products [34]–[36]. Regulatory exceedences of nitrates and herbicide products (e.g. triazines, triazinones, and chloroacetamide) have been commonplace for several years [34]–[37], but recent detections of neonicotinoid contaminants have created new groundwater quality concerns. Beginning in the spring of 2008, two wells had detections of 1.25 and 1.47 µg L^−1^ thiamethoxam in Grant and Sauk Counties, WI (Fig. 1B, Table 1). Subsequent sampling later that season identified six additional locations for a total of 17 independent positive thiamethoxam detections that year. Since these early detections, the WI-DATCP-EQ [17],[18] has repeatedly detected thiamethoxam, imidacloprid, and clothianadin residues at 23 different monitoring well locations over a five-year period (Table 1). Although the sampling effort was not uniformly distributed within the state, neonicotinoid detections often correspond to areas where intensive irrigated agricultural production occurs (Fig. 1A). As an indication of specialty crop production intensity, we used county-level potato abundance to better describe trends in historical neonicotinoid detections. Observed frequency and magnitude of neonicotinoid detections did not consistently correspond to potato abundance (Table 1). Although the contribution of potato production to the observed detections was not clear, regulatory agencies have continued to pursue this interaction by sampling where potato occurs at a high density, specifically the Central Sands and Wisconsin River Valley. Groundwater sampling strategies have provided a useful timeline of non-point source agrochemical pollution events in subsurface water resources. Identifying the origin of pollutants in the state is complicated by the diversity of neonicotinoid registrations, application methods and formulations; currently Wisconsin has 164 different registrations for field, forage, tree fruit, vegetable, turf, and ornamentals crops (6 acetamiprid, 18 clothianadin, 4 dinotefuran, 108 imidacloprid, 1 thiacloprid, 26 thiamethoxam) [38].

### Neonicotinoid Losses and Concentrations in Leachate

**Figure 2:**
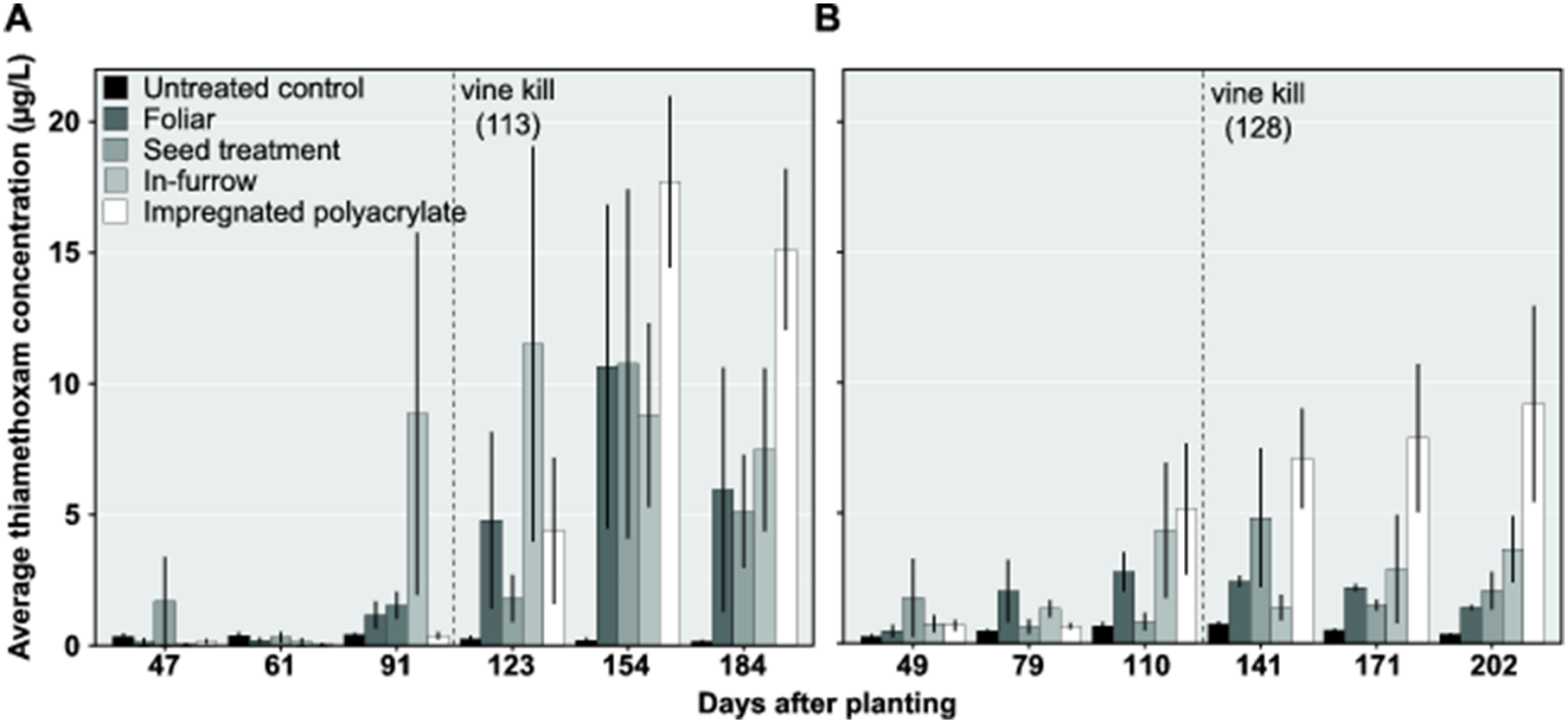
Thiamethoxam concentration in leachate from potato. Average thiamethoxam (±SD) recovered from in-furrow and foliar treatments in (A) 2011 an (B) 2012. Dotted lines indicate the date that the producer applied vine desiccant prior to harvest. Lysimeter studies continued in undisturbed soil following vine kill.

The neonicotinoid insecticide thiamethoxam was included in field experiments to investigate the potential for leaching losses associated with different types of pesticide delivery. Specifically, formulations of thiamethoxam were applied as foliar and as at plant systemic treatments in commercial potato over two years and at two different irrigated fields. We hypothesized that thiamethoxam would be most vulnerable to leaching early in the season when plants were small and episodic heavy rains can be common. Interestingly, we observed the greatest insecticide losses following vine-killing operations which occurred more than 100 days after planting (Fig. 2). Detections of thiamethoxam in lysimeters varied between insecticide delivery treatments through time in 2011 (delivery×date interaction, *F* = 2.1; d.f. = 20,88; *P* = 0.0131) and again in 2012 (delivery×date interaction, *F* = 1.8; d.f. = 20,87; *P* = 0.0384). Moreover, the impregnated polyacrylamide delivery produced the greatest amount of thiamethoxam leachate late in each growing season (Fig. 2) when compared with other types of insecticide delivery.

**Figure 3:**
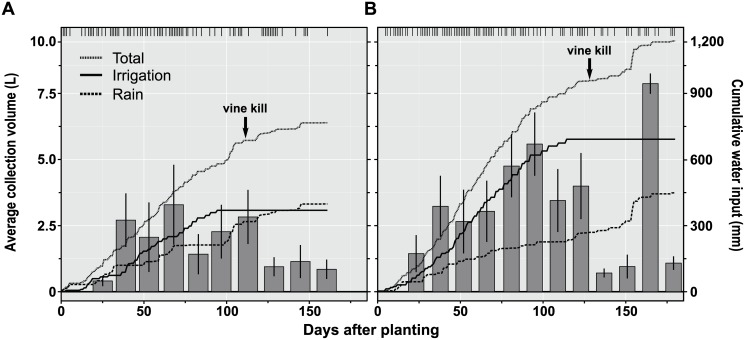
Water input volumes, 2011 and 2012. Water inputs and leachate volume collected in lysimeter studies in (A) 2011 and (B) 2012. Lines indicate cumulative water measured in tipping bucket rain gauges installed in plots each season. Bar plots indicate average leachate volume (±SD) collected in lysimeters on a bi-monthly sampling frequency. Hash marks at the top of each figure indicate days that overhead irrigation or rainfall occurred in each season.

Early season rainfall was not exceptionally heavy in either year of this experiment (Fig. 3). The accumulation of leachate detections in lysimeters likely is reflected by the steady application of irrigation water and rainfall. One clear exception to this pattern occurred in 2012 at 155–156 days after planting when 89 mm of rain fell within a 24-hour period. Peak detections of thiamethoxam in 2012 began to trend upward following this rain event, however the timing of similar detections across treatments in 2011 occurred at about the same time. One additional explanation may be that increased levels of pesticide losses are associated with plant death or senescence. In each year of this study, the largest proportion of pesticide detections in leachate occurred after vine killing with herbicide in the potato crop. Vine killing in commercial potato production is a common practice designed to aid the tubers in developing a periderm. Perhaps the rapid loss in root function following plant death permits excess pesticide to be solubilized and washed through the soil profile more quickly in root channels. In both seasons of this study, however, large episodic rain events did not occur early in the growing season. These results do appear, however, to document low to moderate levels of leaching losses that occur throughout the season even when the crop is managed at nominal evapo-transpirative need.

Untreated control plots also yielded low-level detections of thiamethoxam throughout both seasons. To better understand these insecticide detections in control plots, we sampled water directly from the center pivot irrigation system providing irrigation directly to the potato crop. Samples were taken while the systems were operational from lateral spigots mounted on the well casings. In both years, samples revealed low concentrations of thiamethoxam present in the groundwater at two time points in each sample season (Table 2) from which irrigation water was being drawn. Clothianidin was also present at a single time point in 2012 (Table 2). These positive detections of low-dose thiamethoxam were obviously being unintentionally applied directly to the crop through irrigation and this information is new to the producers in the Central Sands of Wisconsin. Although systemic neonicotinoids have recently been detected from surface water runoff and catch basins associated with irrigated orchards [10], [39], to our knowledge no other study has documented the occurrence of neonicotinoids in subsurface groundwater being recycled through operating irrigation wells. Currently, the known exposure pathways for insecticide residues are most often associated with direct application or systemic movement of insecticides in floral structure and guttation water [8],[9],[40].

**Table 2:** Neonicotinoid concentration from irrigation water, 2011 and 2012.

		Insecticide concentration (µg/L)^a^	
Date	Days after planting	clothianidin	thiamethoxam
28 June 2011	39	-	0.310
1 September 2011	114	-	0.327
10 July 2012	60	-	0.533
15 August 2012	96	0.225	0.580

a Samples obtained from irrigation pivots while under operation in potato fields containing lysimeter experiments.

**Figure 4:**
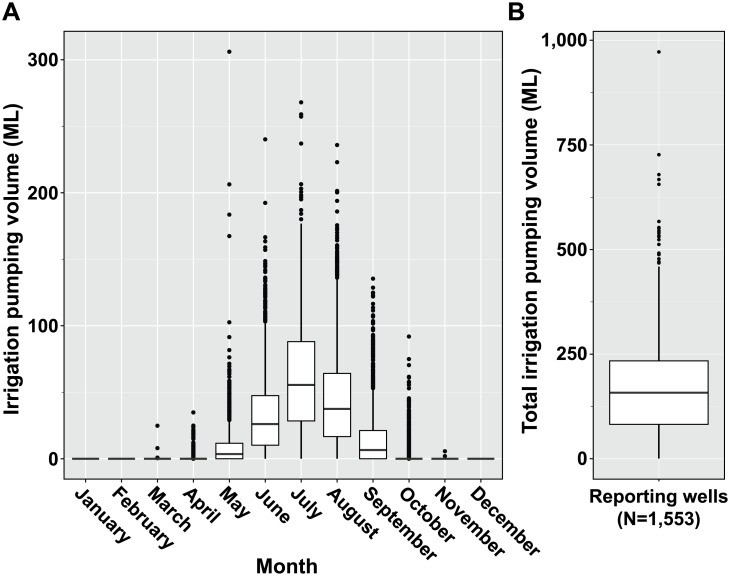
Reported irrigation inputs in the Central Wisconsin River Water Management Unit. Average reported agricultural pumping (megaliters, ML) in the Central Wisconsin River Water Management Unit for 2012. Monthly pumping records were reported by growers to the Wisconsin Department of Natural Resources Bureau of Drinking Water and Groundwater. Upper and lower whiskers extend to the values that are within 1.5*Inter-quartile range beyond the first (25%) and third (75%) percentiles. Data beyond the end of whiskers indicate outlier values and have been plotted as points.

The implications for non-target effects resulting from these groundwater contaminants is currently unknown, but could be important considering the scale of irrigation ongoing in the Central Sands potato agroecosystem in Wisconsin (Fig. S2). Using a combination of aerial photography and NASS Cropland Data Layers, we identified 2,530 different irrigated field units distributed within the Central Wisconsin River Water Management Unit (Fig. S2). In all, 71,864 hectares of irrigated cropland were identified within the extent of the water management unit. Average irrigated field unit size was 28.4±17.7 hectares (min. 1, max 138). Irrigation use patterns demonstrated clear increases in the summer months of the 2012 growing season (Fig. 4). Average annual pumping volume reported to the Wisconsin Department of Natural Resources in 2012 was 170.6±115.6 megaliters (ML) of irrigation water (min. 0.00001, max 972.1) distributed over 1,553 reporting wells. Peak pumping volumes occurred in the month of July, averaging 61±43.3 ML (min. 0, max 286.4). The timing of peak pumping correspond with crop demands for and reproductive phases of common open and closed pollination crops grown in the region.

While considerable attention has been focused on the positive attributes of the neonicotinoids [1]–[3], an increasing body of research suggests substantial negative impacts not only in terms of pest resistance development (e.g., Colorado potato beetle), but also impacts on non-target organisms and surrounding ecosystems [8],[10],[41]–[44]. Recent studies have documented the negative influence of neonicotinoids on pollinator population health (both native and managed) which, in turn, created substantial concern about the long-term sustainability of these pesticides in agriculture [7],[11],[43],[45]–[49]. Exposures to pollinators reportedly occur through chronic, sub-lethal contact with low concentrations of neonicotinoid residues in pollen, nectar, waxes, and guttation drops of common crop plants [50]–[53]. Gill et al. [43] and Whitehorn et al. [54] found that low concentrations (≤10 µg L^−1^) of imidacloprid significantly reduced colony-level health in bumblebee (*Bombus terrestris* L.). Imidacloprid residues measured by those authors are consistent with insecticide concentrations found in nectar and pollen of flowering crops, further supporting the direct crop-pollinator toxicological pathway hypothesis [47],[52],[54],[55]. Though they have received much less attention, many closed pollination crops also provide resources for pollinators (e.g., pollen, water)[56],[57]. These crops also rely on neonicotinoids and may have currently undescribed risks for non-target organisms through indirect contaminant pathways in the agroecosystem [51],[58].

**Figure 5:**
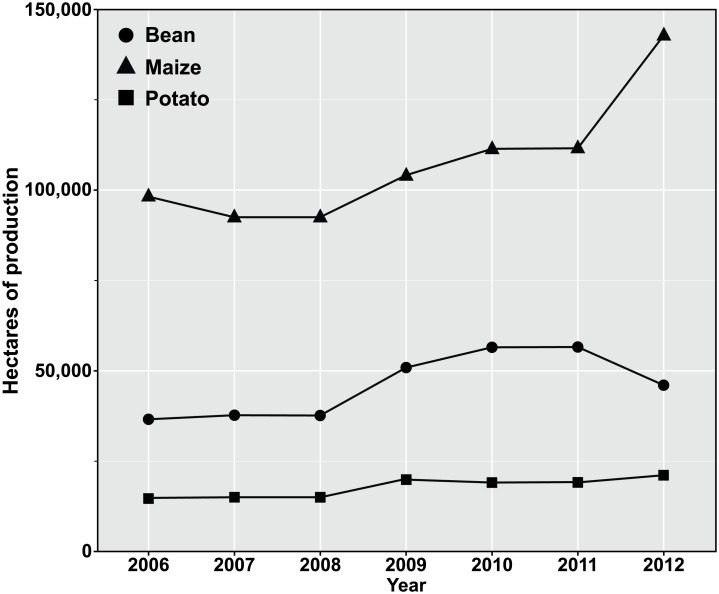
Crop area grown in the Central Wisconsin River Water Management Unit. Cropping trends in the Central Wisconsin River Water Management Unit from 2006–2012. Crop groups are often planted with a soil-applied neonicotinoid insecticide for insect pest management. Crop totals within the water management unit were tabulated from annual USDA-NASS Cropland Data Layers [26].

Possible exposure related to a high frequency of irrigation could drive the exposure of non-target arthropods to low concentrations of neonicotinoid insecticides in irrigation water. Although such impacts have yet to be documented directly, new comprehensive reviews of neonicotinoid environmental impacts have demonstrated numerous unanticipated impacts occurring at the ecosystem scale [9],[58]. In the Wisconsin agroecosystem, neonicotinoids are used on a large proportion of crops grown with irrigation [28],[29]. Trends in production show increased maize production over the past six years in the Central Wisconsin River Water Management Unit (Fig. 5). As a result of common neonicotinoid seed treatment on maize, accelerating production may partially explain the increased frequency of neonicotinoid detection in groundwater. Unfortunately, little crop-specific pesticide information exists for individual neonicotinoids at the watershed scale [26]. Although measurement of specific contributions of crops to measured insecticide contamination is currently not available, this study demonstrates a research approach to better understand leaching from different application methods. Improved understanding of crops and insecticide delivery that results in greater risk of insecticide leaching will inform targets to reduce aquifer contamination and recirculation of soil-applied insecticides. Area-wide application of neonicotinoid insecticides through irrigation water applications may have considerable unanticipated or undocumented environmental impacts for non-target organisms through chronic low-dose exposure to insecticides.

## Conclusions

To gain a better understanding of the seasonal cycle of neonicotinoids moving from the potato system, this study used an experimental approach to document the leaching potential of common neonicotinoid application methods. Results presented here benefit both potato producers and regulators by identifying trends in leachate losses for these commonly used, water-soluble insecticides. Lysimeter experiments documented loss of thiamethoxam following the application of vine desiccants at the conclusion of the potato production season. Leachate losses did vary among the different delivery methods over time indicating some variability in the patterns of pesticide leachate throughout the season. Quantification of crops commonly using neonicotinoid soil applications in the Central Wisconsin Water Management Unit highlights the need to research leaching potential from soil-applied neonicotinoids in other commodities. Documentation of several neonicotinoids in irrigation water suggests a new candidate pathway for non-target environmental impacts of insecticides.

## Supporting Information

Figure S1 **Chemical structures and properties of common neonicotinoid insecticides.** Chemical structures were drawn using ChemDraw (version 13, Perkin Elmer Inc., Waltham, MA). Properties of each active ingredient were accessed from the National Center for Biotechnology Information PubChem online interface. Available: https://pubchem.ncbi.nlm.nih.gov/. Accessed 2014 Mar 20.
(TIF)

Figure S2 **Irrigated field locations in the Central Wisconsin River Water Management Unit.** Distribution of fields irrigated with high capacity wells (n = 2530) in the Central Wisconsin River Water Management Unit [27]. Points indicate locations of individual irrigation units identified from aerial photography using ArcGIS.
(TIF)
