# Supplementary figures and images for: Environmental Fate of Soil Applied Neonicotinoid Insecticides in an Irrigated Potato Agroecosystem
Source: PLoS One. 2014 May 13;9(5):e97081. doi: 10.1371/journal.pone.0097081 (PMC4019649; doi:10.1371/journal.pone.0097081)

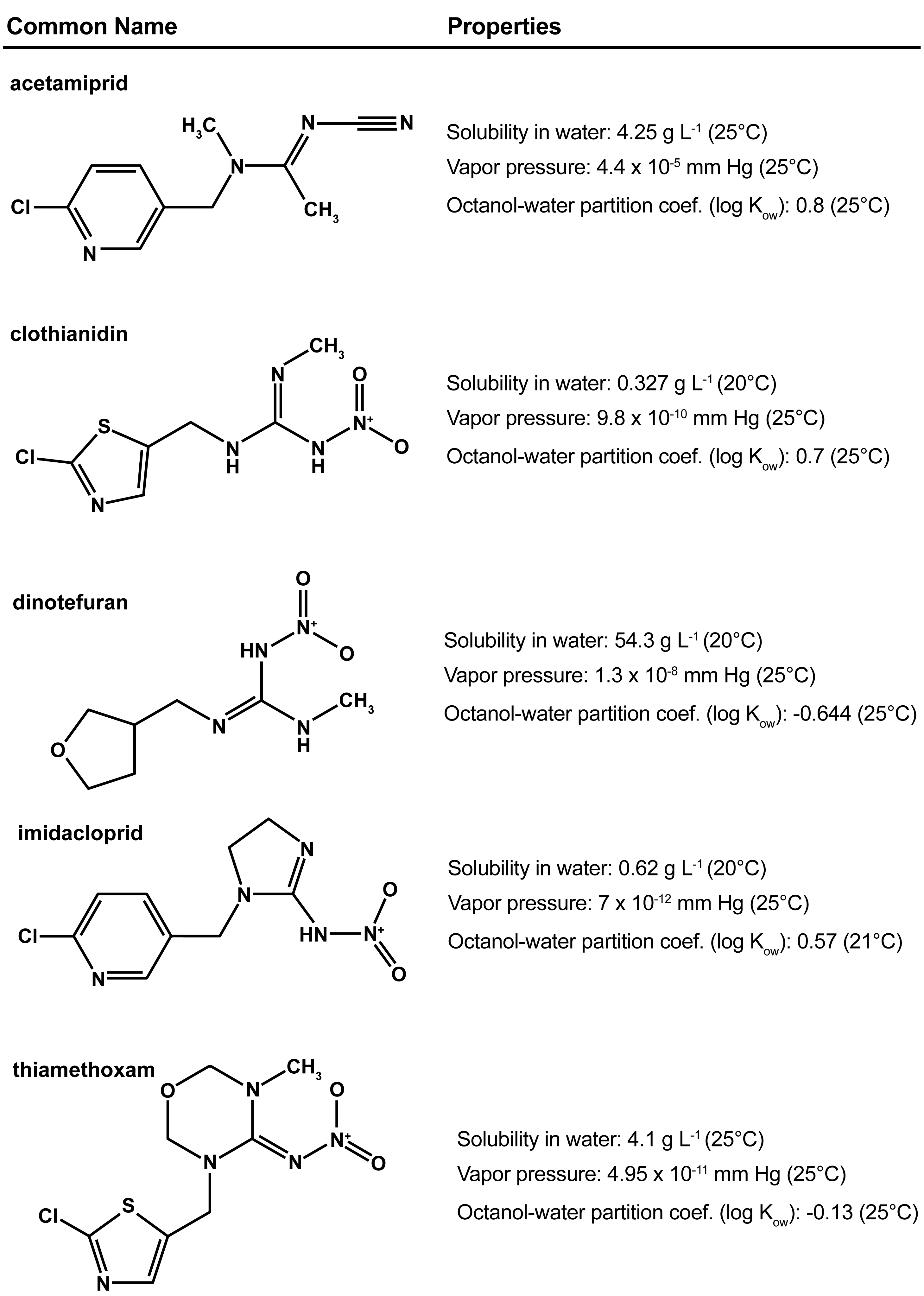

Supplement: Figure S1 — Chemical structures and properties of common neonicotinoid insecticides. Chemical structures were drawn using ChemDraw (version 13, Perkin Elmer Inc., Waltham, MA). Properties of each active ingredient were accessed from the National Center for Biotechnology Information PubChem online interface. Available: https://pubchem.ncbi.nlm.nih.gov/. Accessed 2014 Mar 20. (TIF) [file pone.0097081.s001.tif]

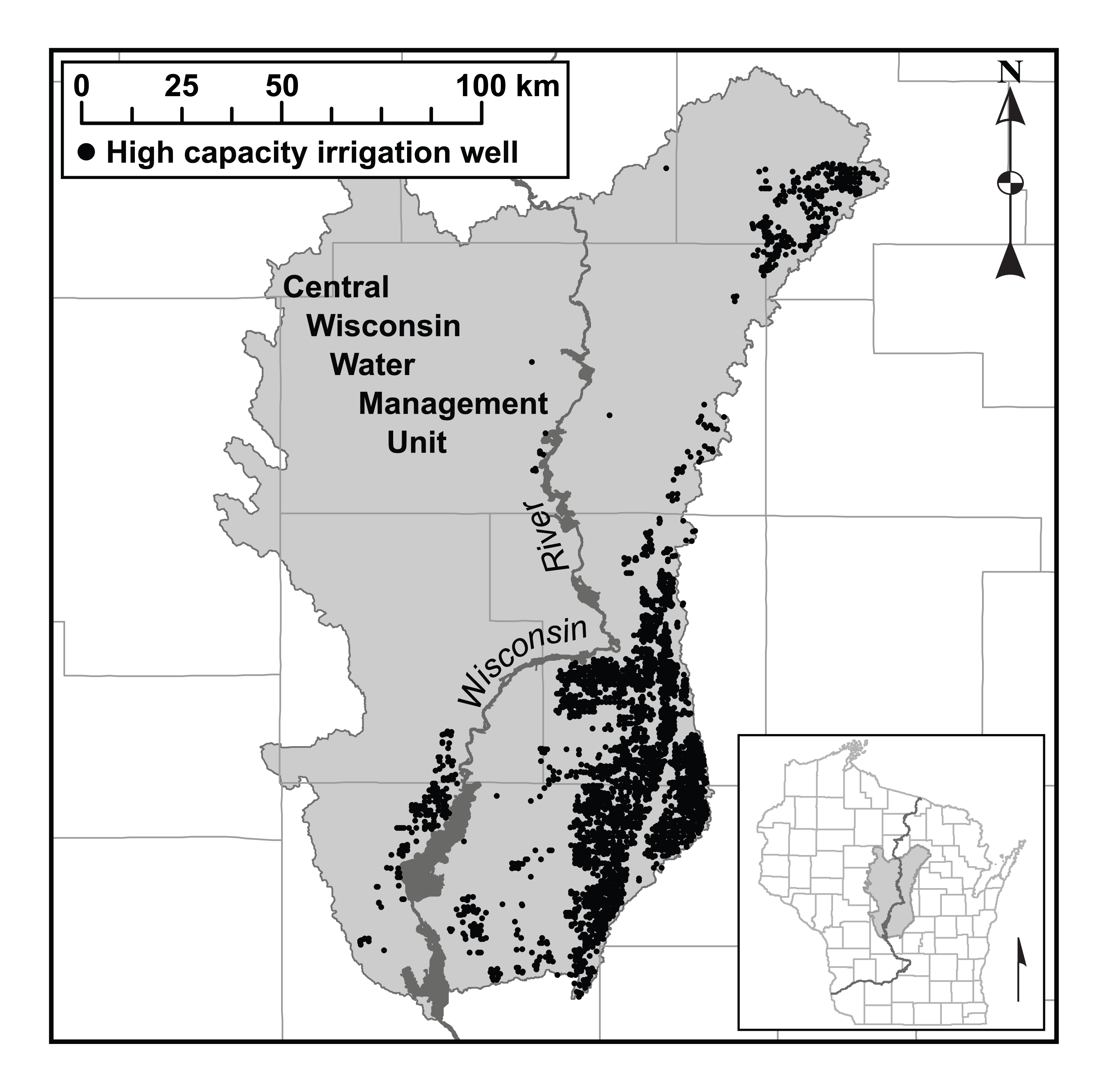

Supplement: Figure S2 — Irrigated field locations in the Central Wisconsin River Water Management Unit. Distribution of fields irrigated with high capacity wells (n = 2530) in the Central Wisconsin River Water Management Unit [27]. Points indicate locations of individual irrigation units identified from aerial photography using ArcGIS. (TIF) [file pone.0097081.s002.tif]
